# 322. Personal and Environmental Decolonization Strategies for Children with Staphylococcus aureus SSTI and Their Households – A Randomized Clinical Trial

**DOI:** 10.1093/ofid/ofaf695.111

**Published:** 2026-01-11

**Authors:** Alaina Schneider, Mary G Boyle, Lisa Richardson, Stephanie A Fritz

**Affiliations:** Washington University School of Medicine, St. Louis, Missouri; Washington University School of Medicine, St. Louis, Missouri; Washington University, St. Louis, Missouri; Washington University School of Medicine, St. Louis, Missouri

## Abstract

**Background:**

Recurrent *Staphylococcus aureus* skin and soft tissue infections (SSTI) pose a significant burden. Decolonization with topical antimicrobials reduces, but does not eliminate, this burden. The household environment is a reservoir for *S. aureus* transmission. Thus, we hypothesized that targeting both personal and household environmental *S. aureus* colonization would reduce recurrent SSTI incidence.

**Figure:**
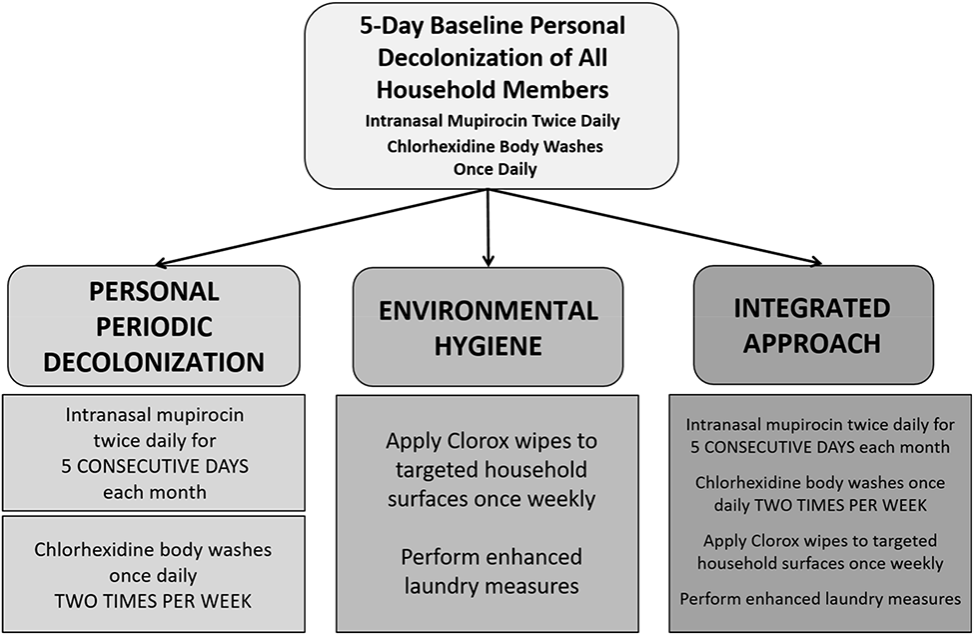
SHINE Trial randomization scheme.

**Figure:**
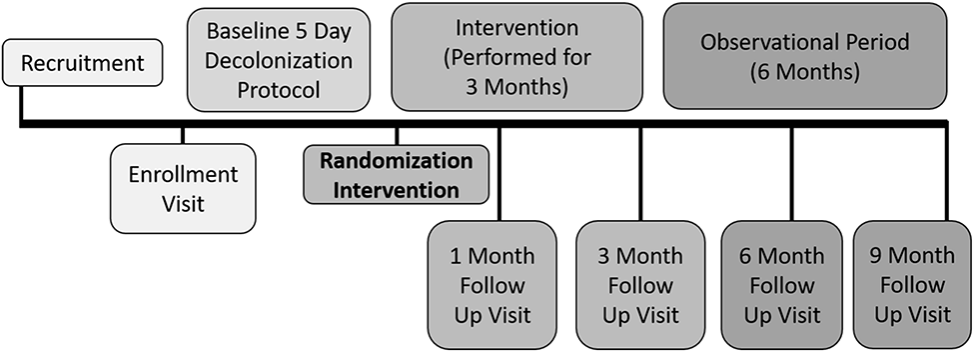
SHINE Trial timeline

**Methods:**

“*Staph* Household Intervention for Eradication (SHINE)” was a pragmatic, open-label randomized trial, enrolling children with community-associated *S. aureus* SSTI and their household contacts. All household members were assigned a 5-day decolonization regimen (Figure 1). Households were then randomized 1:1:1 to Personal Periodic Decolonization, Environmental Hygiene, or an Integrated Approach of both Personal Periodic Decolonization and Environmental Hygiene (Figure 1). At 5 study visits over 9 months, household members and environmental surfaces were sampled to detect *S. aureus* colonization (Figure 2). SSTI incidence was recorded for all household members.

**Figure:**
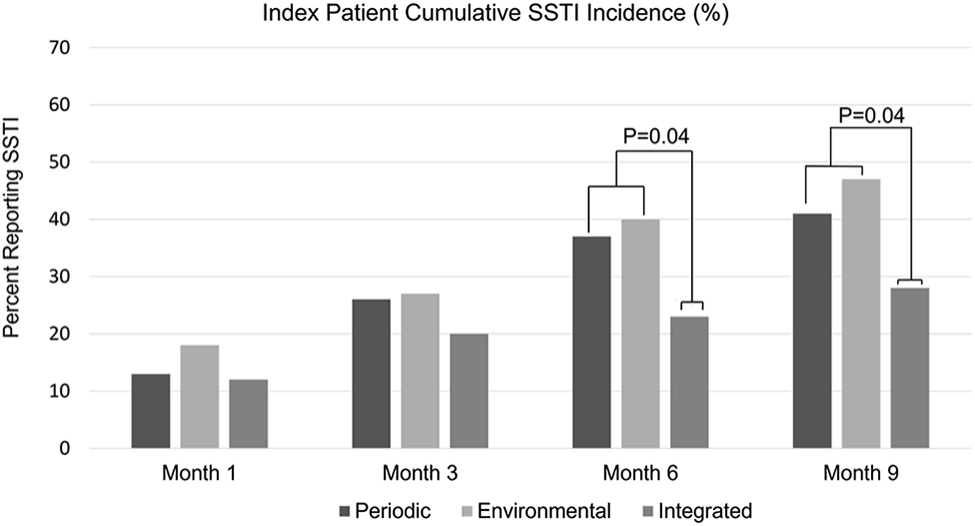
Cumulative SSTI incidence reported by index patients at each longitudinal study visit by assigned intervention. Cumulative SSTI incidence was significantly lower for the Integrated Approach group compared to the combined Personal Periodic and Environmental Hygiene groups at 6-months (p=0.04) and 9-months (p=0.04).

**Figure:**
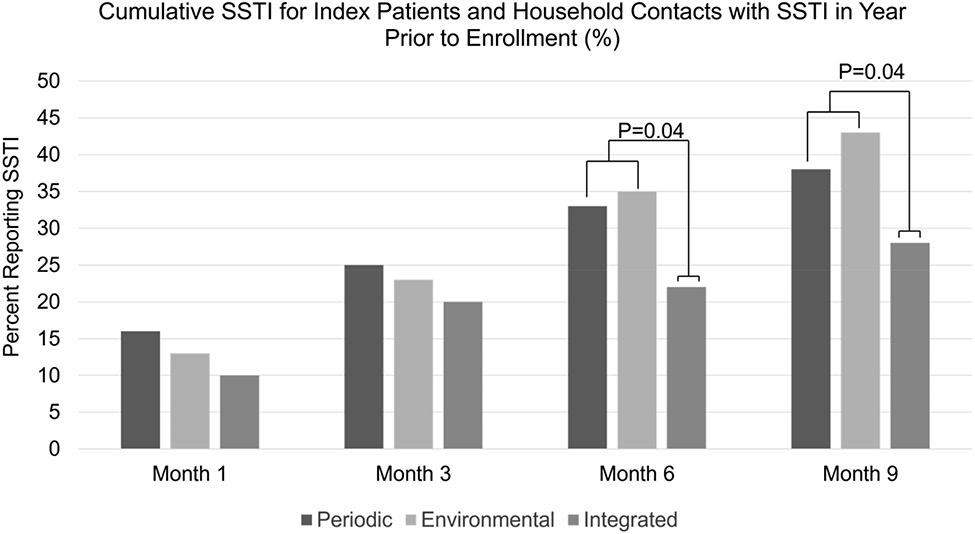
Cumulative SSTI incidence for index patients and household contacts with SSTI in the year prior to study enrollment combined. Participants with SSTI in the year prior to trial enrollment assigned to the Integrated Approach had a lower cumulative SSTI incidence at 6-months (p=0.04) and 9-months (p=0.04), compared to the combined Personal Periodic and Environmental Hygiene groups.

**Results:**

196 index patients and their 639 household contacts were enrolled. 56% of participants were female; 64% were Caucasian and 36% were African American or multiracial. 28% had experienced an SSTI in the year before trial enrollment. 69% of the index patients had an SSTI caused by methicillin-resistant *S. aureus* (MRSA) and 31% by methicillin-susceptible *S. aureus* (MSSA). Overall, cumulative SSTI incidence in index patients was 14% 1 month following enrollment, 24% at 3 months, 33% at 6 months, and 39% at 9 months. Cumulative SSTI incidence did not differ between intervention groups during the 3-month intervention. Among index patients and household contacts with SSTI in the prior year (Figures 3 and 4), SSTI incidence was lower among the Integrated Approach at 6- and 9-months compared to the other groups. In multivariable models, history of SSTI in the year prior to study enrollment was a significant predictor of SSTI incidence during the trial period.

**Conclusion:**

While an Integrated Approach of prolonged personal decolonization and environmental hygiene reduced SSTI among participants with prior year SSTI, prior SSTI was the strongest predictor of longitudinal SSTI. Novel prevention strategies are needed.

**Disclosures:**

All Authors: No reported disclosures

